# Generational Differences in Support and Care of Individuals Diagnosed With Severe Mental Illness

**DOI:** 10.1093/geroni/igaa057.310

**Published:** 2020-12-16

**Authors:** Deborah Finkel, Per Bulöw, Pia Bulöw, Monika Wilińska, Cristina Joy Torgé, Marie Ernsth Bravell, Magnus Jegermalm

**Affiliations:** 1 Indiana University Southeast, New Albany, Indiana, United States; 2 Jönköping University, Jönköping, Sweden

## Abstract

As part of the process of de-institutionalizing the Swedish mental health care system, a reform was implemented in 1995 moving responsibility for social support for people with severe mental illness (SMI) from the county to social services in the municipalities. In many ways, older people with SMI were neglected in this changing landscape of psychiatric care. To investigate possible generational differences in support experiences, two nonoverlapping cohorts were created from surveys conducted every fifth year between 1996 and 2011 in one middle-sized municipality in the south of Sweden, aiming to detect the needs for social support. Cohort 1 includes everyone detected at the 1996 survey aged 65 and 79 years (N = 92). Cohort 2 includes individuals first detected at the 2011 survey who were aged 65 to 79 (N = 104). Results indicates significant differences between the two cohorts in diagnosis, reflecting changes over time in diagnostic tendencies. Cohort 1 was on average 10 years older than Cohort 2, even within the restricted age range. After correcting for age, there were no differences between the two cohorts in education, functioning (CAN and GAF), or marital status. Although Cohort 1 experienced more days of institutionalization than Cohort 2 (median = 424.5 days vs. 382 days), the difference was not statistically significant. Cohort 2 had significantly higher additional subsidies and disposable income, as well as significantly higher income from other sources after retirement. Results indicate the changing demands that older adults with SMI will place on care systems.

